# Restless legs syndrome augmentation among Japanese patients receiving pramipexole therapy: Rate and risk factors in a retrospective study

**DOI:** 10.1371/journal.pone.0173535

**Published:** 2017-03-06

**Authors:** Masayoshi Takahashi, Shingo Nishida, Masaki Nakamura, Mina Kobayashi, Kentaro Matsui, Eiki Ito, Akira Usui, Yuichi Inoue

**Affiliations:** 1 Department of Somnology, Tokyo Medical University, Tokyo, Japan; 2 Japan Somnology Center, Neuropsychiatric Research Institute, Tokyo, Japan; 3 Maezawa Hospital, Tochigi, Japan; 4 Department of Psychiatry, Tokyo Women’s Medical University, Tokyo, Japan; University of Rome Tor Vergata, ITALY

## Abstract

To investigate the rate of and risk factors for restless legs syndrome (RLS) augmentation in Japanese patients receiving pramipexole (PPX) treatment. Records of 231 consecutive patients with idiopathic RLS who received PPX therapy for more than one month in a single sleep disorder center were analyzed retrospectively. Augmentation was diagnosed based on the Max Planck Institute criteria; associated factors were identified by logistic regression analysis. Mean age at PPX initiation was 60.6 ± 14.9 years and mean treatment duration was 48.5 ± 26.4 months. Augmentation was diagnosed in 21 patients (9.1%). Daily PPX dose and treatment duration were significantly associated with augmentation. By analyzing the receiver operating characteristic curve, a PPX dose of 0.375 mg/day was found to be the optimal cut-off value for predicting augmentation. After stratifying patients according to PPX treatment duration, at median treatment duration of 46 months, optimal cut-off values for daily doses were 0.375 and 0.500 mg/day for <46 months and ≥46 months of treatment, respectively. The RLS augmentation with PPX treatment in Japanese patients was occurred at rate of 9.1%, being quite compatible with previously reported rates in Caucasian patients. The symptom could appear within a relatively short period after starting the treatment in possibly vulnerable cases even with a smaller drug dose. Our results support the importance of keeping doses of PPX low throughout the RLS treatment course to prevent augmentation.

## Introduction

Restless legs syndrome (RLS) / Willis—Ekbom disease is a sensorimotor disorder with leg discomfort and the irresistible urge to move affected body parts. Dopaminergic drugs are widely accepted to be effective for the treatment of RLS. However, long-term treatment with this drug type may cause augmentation, an aggravated state of RLS symptoms including an earlier onset of the symptom or an expansion to other body parts, compared with the initial period of dopaminergic treatment [1]. Most patients with augmentation also have a paradoxical response to pharmacological treatment or have shorter duration of the treatment effect than during early treatment days. Although it has been suggested that the rate of augmentation increases with longer duration of dopaminergic treatment or with higher doses of this drug type [2], the risk factors for augmentation have not been conclusively identified.

The rate of augmentation has been estimated at 14.2% to 73% in RLS patients taking L-dopa and also occurs to some extent in patients using dopamine agonists (DAs) [3]. As for pramipexole (PPX), a non-ergot short acting DA widely used as a first-line treatment for RLS, it has been reported that, in Europe and the United States, 8.3% to 70% of RLS patients develop augmentation during a long term course of treatment with the drug [4–10]. However, there have been very few reports on augmentation in Asian countries [11], and conclusive information about the prevalence of augmentation in patients residing in this region has not been obtained. Considering these issues, we initiated this study to investigate the rate of augmentation and factors associated with the presence of the symptom in Japanese RLS patients receiving PPX therapy.

## Materials and methods

The study was approved by the Ethics Committee for Human Research of the Neuropsychiatric Research Institute (Tokyo, Japan). All patients included in this study gave written informed consent for the participation at the first visit. In this retrospective study, 275 consecutive outpatients were eligible who had been diagnosed with RLS according to NIH/IRLSSG criteria [12] at Yoyogi Sleep Disorder Center (Tokyo, Japan) and who had been regularly using PPX for the treatment of the disorder for more than one month between September 2004 and May 2013. The patients who had any background conditions causative for secondary RLS (e.g., iron deficiency, pregnancy, cardiovascular or cerebrovascular disease, renal failure, peripheral neuropathy, multiple sclerosis) were not included in the 275 patients. As for iron deficiency, the patients, who had deficient condition indicated by laboratory test and whose RLS symptoms were not improved after correction of serum ferritin level by iron supplementation, were diagnosed as idiopathic RLS and included in the subject of the present study. After thorough reviews of the medical records, 44 were excluded from the study due to the following reasons: receiving DAs other than PPX (n = 18); receiving concurrent medications which may worsen RLS symptoms [13] (antidepressants, n = 9); discontinuation of PPX treatment through the treatment course (n = 9); RLS mimics [13,14] (n = 3); and intermittent RLS [13] (n = 5). The patients were included who used concomitant medications for RLS (benzodiazepines, iron supplementation, α2δ ligands) which were reported not to cause augmentation [15]. Thus, data from 231 patients were subjected to analyses in this study.

The descriptive data (sex, age, body mass index (BMI), presence/absence of family history of RLS, severity of RLS at the start of PPX treatment, the daily dose and treatment duration of PPX, presence/absence of prior treatment for RLS with dopaminergic drugs or concomitant medications for RLS other than DA) were collected from the medical records.

The PPX were administered at the initial dose of 0.125 mg/day. The doses of PPX were increased or decreased according to patients’ status at regular visits. If RLS symptom were sufficiently improved and stable without side effects, the daily PPX doses were fixed and thereafter unchanged. However, when a symptom suspected to be clinically relevant augmentation symptom occurred, the dose of PPX was decreased or the drug was switched to alternative drugs (longer-acting DAs or α2δ ligands) if necessary. For this reason, in patients having episodes of augmentation, the daily PPX doses at the onset of augmentation were used for the analyses. The time to the onset of augmentation (cases with augmentation) or time to the final observation (cases without augmentation) from the start of PPX treatment was also used as the treatment duration of PPX. The severity of RLS at the start of PPX treatment was assessed using the total score of the Japanese version of the international restless legs syndrome study group rating scale (IRLS) [16]. The attending physicians (all of them were board-certified sleep disorder-experts) reported the suspected augmentation symptoms in the medical records, such as onset time of symptoms, latency of symptoms at rest, affected body parts, subjective intensity of the symptoms or duration of relief after PPX medication. Paradoxical response to changes in PPX dose were also recorded when the suspected augmentation symptoms occurred. The final diagnosis of augmentation was based on the retrospective reviews of the medical records according to the Max Planck Institute diagnostic criteria [1]. If the symptoms were not clearly diagnosed as augmentation (n = 2), the suspected patients were reassessed by another sleep disorder-expert physician, and the diagnosis was made based on discussion between them. The severity of augmentation was assessed retrospectively using the augmentation severity rating scale (ASRS) total score [17] based on the review of the medical records. In most cases affected with augmentation, the serum ferritin level was also determined at the onset of augmentation.

Logistic regression analyses to identify factors associated with the presence of augmentation were performed with independent variables including sex, age, BMI, IRLS total score at the start of PPX treatment, treatment duration of PPX (as a continuous variable or a categorical variable dichotomized by median treatment duration), daily PPX dose, presence/absence of prior DA treatment for RLS, and presence/absence of concomitant medications for RLS (benzodiazepines, iron supplementation or α2δ ligands). Univariate and multivariate logistic regression analyses were performed to calculate odds ratios, and the Wald test was used to assess the significance of each variable. Additionally, predictive daily PPX dose for the occurrence of augmentation was assessed using receiver operating characteristic (ROC) curve analysis. In this analysis, optimal cut-off value (Youden index method), the area under the curve (AUC), sensitivity, specificity, positive predictive value (PPV), negative predictive value (NPV), positive likelihood ratio (LR+) and negative likelihood ratio (LR−) were calculated. Statistical significance was set at p < 0.05 (two-tailed). All analyses were performed using the R statistical package (version 3.0.2).

## Results

Among 231 patients, the male/female ratio (%) was 40.7/59.3, age at the start of PPX treatment (average ± SD) was 60.6 ± 14.9 years, IRLS total score at the start of the treatment was 24.0 ± 6.3, the treatment duration of PPX was 48.5 ± 26.4 months (range 2–104 months), the daily PPX dose was 0.262 ± 0.119 mg/day (range 0.031–0.750 mg/day), and 18 patients (7.8%) had received prior treatment with dopaminergic drugs for RLS (L-dopa, n = 2; non-ergot DAs, n = 8; ergot DAs, n = 8). One hundred sixty one patients (69.7%) had concomitant medications for RLS other than DAs (Table 1).

**Table 1:** Descriptive variables of the subjects.

	All patients (n = 231)
Male/Female, % (n)	40.7%/59.3% (94/137)
Age, years^a^	60.6 ± 14.9 (231)
BMI, kg/m^2^	22.3 ± 3.3 (231)
Family history, Yes / No, % (n)	24.3%/75.7% (56/174)
IRLS total score^b^	24.0 ± 6.3 (197)
Treatment duration of PPX, months^c^	48.5 ± 26.4 (231)
Dose of PPX, mg/day^d^	0.262 ± 0.119 (231)
Prior DA treatment for RLS, Yes / No^e^, % (n)	7.8%/92.2% (18/213)
Concomitant medication for RLS, Yes / No, % (n)	69.7%/30.3% (161/70)
Benzodiazepines, Yes / No, % (n)	52.8%/47.2% (122/109)
Iron supplementation, Yes / No, % (n)	25.5%/74.5% (59/172)
α2δ ligands, Yes / No, % (n)	16.0%/84.0% (37/194)

BMI, body mass index; IRLS, International Restless Legs Syndrome Study Group rating scale; PPX, pramipexole; DA, dopaminergic agonist.

Values are Mean ± SD (n) unless otherwise noted.

^a^Age at the start of PPX treatment.

^b^IRLS at the start of PPX treatment (Missing data: n = 34).

^c^In patients with augmentation, time to the onset of augmentation from the start of PPX treatment is shown. In patients without augmentation, time to the final observation from the start of PPX treatment is shown.

^d^In patients with augmentation, dose of PPX at the onset of augmentation is shown. In patients without augmentation, dose of PPX at the final observation is shown.

^e^Patients who had not received previous dopaminergic therapy for RLS.

After thorough review of the medical records by sleep disorder-expert physicians, 21 patients (9.1%) were judged to have episodes of augmentation. All of these episodes were considered to be clinically relevant because the patients who reported an increase in subjective feelings of discomfort and required certain changes of the PPX treatment regimen, including dose reduction or split administration of the drug and switching to other drugs. The ASRS total score at the period of maximal augmentation in these patients was 9.4 ± 3.0 points (range 5–16). The serum ferritin level was measured in 20 patients (95.2%) at the onset of augmentation, and the value was 69.3 ± 54.3 ng/mL (≥ 50 ng/mL, n = 10; < 50 ng/ml, n = 10).

The daily PPX dose, the treatment duration as a categorical variable, the presence of benzodiazepines and the presence of 2δ ligands were significantly associated with the presence of augmentation on univariate analysis (p < 0.001, p = 0.04, p = 0.01 and p = 0.001, respectively) and iron supplementation tended to be associated with the augmentation (p = 0.06) (Table 2). Meanwhile, only the daily PPX dose and the treatment duration were significantly associated with the presence of augmentation on multivariate regression analysis (p < 0.001 and p = 0.001, respectively). When multivariate logistic regression analysis was performed using the treatment duration as a continuous variable, associations of the treatment duration to the presence of augmentation remained significant (p = 0.001). However, other descriptive variables did not appear as significant factors on multivariate models.

**Table 2:** Analysis of factors for association with the presence of augmentation.

Factors		Univariate		Multivariate	
	n	Odds Ratio (95% CI)	p-value^f^	Odds Ratio (95% CI)	p-value^g^
Male	231	1.10 (0.45, 2.73)	0.83	0.56 (0.11, 2.78)	0.48
Age, years^a^	231	1.02 (0.99, 1.06)	0.24	1.04 (0.98, 1.10)	0.16
BMI, kg/m^2^	231	1.07 (0.95, 1.22)	0.27	1.08 (0.88, 1.32)	0.47
IRLS total score^b^	197	1.04 (0.96, 1.12)	0.32	0.96 (0.85, 1.07)	0.44
Treatment duration of PPX, ≥ 46 months / < 46 months^c^	231	0.36 (0.13, 0.96)	0.04	0.06 (0.01, 0.34)	0.001
Daily PPX dose^d^	231	3.48 (2.07, 5.86)	< 0.001	7.87 (3.07, 20.17)	< 0.001
Prior dopaminergic treatment, Yes / No^e^	231	3.29 (0.98, 11.12)	0.06	2.38 (0.31, 18.54)	0.41
Benzodiazepines, Yes/No^f^	231	4.25 (1.38, 13.06)	0.01	5.05 (0.94, 27.23)	0.06
Iron supplementation, Yes/No^f^	231	2.40 (0.96, 6.03)	0.06	3.90 (0.98, 15.47)	0.05
α2δ ligands, Yes/No^f^	231	4.87 (1.88, 12.62)	0.001	3.55 (0.84, 15.01)	0.09

BMI, body mass index; IRLS, International Restless Legs Syndrome Study Group rating scale; PPX, pramipexole; DA, dopaminergic agonist; CI, confidence interval.

^a^Age at the start of PPX treatment.

^b^IRLS at the start of PPX treatment.

^c^In patients with augmentation, time to the onset of augmentation from the start of PPX treatment is shown. In patients without augmentation, time to the final observation from the start of PPX treatment is shown.

^d^In patients with augmentation, dose of PPX at the onset of augmentation is shown. In patients without augmentation, dose of PPX at the final observation is shown. Odds ratios for daily PPX dose expressed as per 0.125 mg increments.

^e^Patients who had not received previous dopaminergic therapy for RLS.

^f^Concomitant medication for RLS.

^g^Wald’s test.

**Fig 1:**
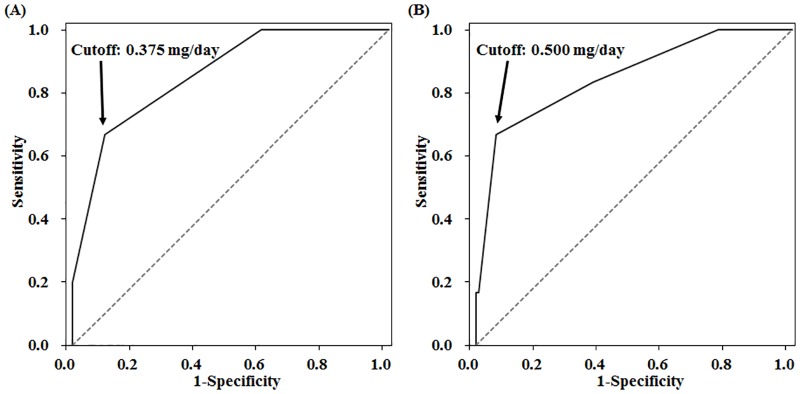
Sensitivity and specificity of the dose of pramipexole for occurrence of augmentation. (A) ROC curve in the patients with < 46 months of PPX treatment duration (n = 114). The optimal cut-off value of daily PPX dose to predict the presence of augmentation was 0.375 mg/day (AUC, 0.860; sensitivity, 66.7%; specificity, 89.9%; PPV, 0.500; NPV, 0.947; LR+, 6.60; LR−, 0.371). (B) ROC curve in the patients with ≥ 46 months of PPX treatment duration (n = 117). The optimal cut-off value of daily PPX dose to predict the presence of augmentation was 0.500 mg/day (AUC, 0.851; sensitivity, 66.7%; specificity, 93.7%; PPV, 0.364; NPV, 0.981; LR+, 10.57; LR−, 0.356). AUC, Area Under the Curve; PPV, Positive Predictive Value; NPV, Negative Predictive Value; LR+, Positive Likelihood Ratio; LR−, Negative Likelihood Ratio.

By ROC curve analysis on the total number of patients, the optimal cut-off value of daily PPX dose to predict the presence of augmentation was 0.375 mg/day (AUC, 0.811; sensitivity, 71.4%; specificity, 75.7%; PPV, 0.227; NPV, 0.964; LR+, 2.94; LR−, 0.377). Since the treatment duration also appeared as a significantly associated factor on multiple logistic regression analysis, we further performed the ROC curve analyses after stratifying the patients into two categories at the median value of treatment duration (46 months). As a result, the optimal cut-off values were 0.375 mg/day and 0.500 mg/day in the patients with < 46 months and with ≥ 46 months of treatment duration, respectively (Fig 1).

## Discussion

To our knowledge, this is the first study reported to estimate the rate of occurrence of augmentation and its associated factors in Asian RLS patients receiving PPX therapy. Although the mean daily PPX dose in the present study was a little lower than those in previously reported studies [4–11], the number of RLS patients targeted for the analyses and their PPX treatment duration in the present study were quite comparable to those in previous studies.

In the present study, the rate of augmentation symptoms was 9.1% in the Japanese RLS patients, which was quite the same as the rate of 8.3% reported by Ferini-Strambi [4] or 9.2% by Hogl [6].

It has been inconclusive whether the rate of augmentation increases along with the increase in daily doses of dopaminergic drugs or not [2,4]. With regard to this, the present study revealed that the daily PPX dose appeared as an associated factor for the occurrence of augmentation, supporting that the doses of DAs is important factor to develop augmentation. To our knowledge, there have been no studies showing the cut-off of daily PPX dose for predicting the presence of augmentation. Interestingly, ROC curve analysis on the total patient group showed that the daily PPX dose of 0.375 mg/day was the optimal cut-off value for predicting the occurrence of the symptom. With regard to this, in previous studies showing much higher rates of augmentation during PPX treatment (between 32% and 70%) [8–11], the mean daily PPX dose at final observation of the studied patients was clearly higher (ranging between 0.63 and 1.28 mg/day) than that of the patients analyzed in the present study. Considering these things, we want to emphasize that the daily PPX dose should be kept low for the prevention of augmentation.

Of note, in contrast to the accepted notion that the appearance of augmentation becomes frequent along with prolongation of treatment duration with dopaminergic drugs [2], in the present study, a relatively shorter treatment duration of PPX (< 46 months) appeared as a significantly associated factor with the presence of augmentation by logistic regression analysis among the subject Japanese RLS patients. Besides, in the patients with < 46 months of treatment, the optimal cut-off value of PPX was lower than that in patients with ≥ 46 months (0.375 mg/day vs. 0.500 mg/day). Although it is unclear whether this phenomenon is a racial characteristic of Japanese RLS patients, it is possible that a certain number of individuals affected with RLS are vulnerable to development of augmentation with smaller doses of dopaminergic drugs within relatively short periods after starting treatment.

There are a few limitations of the present study. First, this study was conducted as a retrospective review of the medical records. Hence, mild augmentation symptoms without clinical relevance might have been overlooked by attending physicians. In addition, the augmentation severity rating scale has not been validated for retrospective use. Second, the serum ferritin levels were measured only when augmentation occurred and were not measured in patients without augmentation. A low ferritin level has been reported as a risk factor for developing augmentation [18,19], and the present study showed that half of the patients with augmentation who received serum ferritin measurements had a relatively low level (< 50 ng/ml). However, whether low serum ferritin was associated with the presence of augmentation could not be determined, because serum ferritin measurement was not conducted in patients without augmentation. Third, although the combination of PPX and other DAs might further increase the occurrence of augmentation, we did not assess the rate of augmentation in patients receiving the combination therapy due to a scarcity of this kind of the patients. Fourth, since this study was conducted in a single sleep disorder center, our sample might not be representative of the general RLS population in Japan. Finally, we could not identify the causal relationship between the occurrence of augmentation and the associated factors with this retrospective study.

In conclusion, we found that augmentation with PPX treatment was 9.1% in Japanese RLS patients which was quite the same as the previously reported rates in Caucasian patients, and that the symptoms could appear within a relatively short period after starting the treatment in possibly vulnerable cases using a small dose of the drug. Our results support the importance of keeping doses of DAs low throughout the RLS treatment course to prevent augmentation. Future prospective placebo-controlled multi-center studies would be necessary to confirm the results of the present study.

## Supporting information

S1 Table The individual-level data. (DOC)
