# Supplementary material for: Restless legs syndrome augmentation among Japanese patients receiving pramipexole therapy: Rate and risk factors in a retrospective study
Source: PLoS One. 2017 Mar 6;12(3):e0173535. doi: 10.1371/journal.pone.0173535 (PMC5338826; doi:10.1371/journal.pone.0173535)
Supplement: S1 Table — (DOC) [file pone.0173535.s001.doc]

S1 Table. The individual-level data.

| No. | Gender | Age | BMI | IRLS  Total score | Dose  of PPX | Augmen-  tation | Treatment  Duration  of PPX | Family  history | Prior DA  Treatment  for RLS | Concomitant medication | | |
| --- | --- | --- | --- | --- | --- | --- | --- | --- | --- | --- | --- | --- |
|  |  |  |  |  |  |  |  |  |  | Benzodiaze-  pines | Iron  supplementation | a2d ligands |
| 1 | Male | 37 | 19.3 | 30 | 0.675 | Yes | 36 | No | Yes | Yes | No | No |
| 2 | Female | 62 | 25.6 | 32 | 0.375 | Yes | 2 | No | Yes | Yes | Yes | Yes |
| 3 | Male | 79 | 24.3 | 18 | 0.5 | Yes | 55 | Yes | Yes | No | Yes | Yes |
| 4 | Male | 69 | 25.1 | 37 | 0.375 | Yes | 16 | No | No | Yes | No | No |
| 5 | Female | 65 | 22 | 17 | 0.375 | Yes | 73 | No | No | Yes | No | No |
| 6 | Female | 70 | 24.3 | 21 | 0.25 | Yes | 69 | No | No | Yes | Yes | No |
| 7 | Male | 53 | 24.3 | 24 | 0.75 | Yes | 71 | No | No | Yes | No | No |
| 8 | Male | 64 | 25.7 | 28 | 0.375 | Yes | 33 | No | No | Yes | Yes | Yes |
| 9 | Male | 55 | 23.3 | 32 | 0.5 | Yes | 64 | No | Yes | Yes | No | Yes |
| 10 | Male | 65 | 22.3 | 33 | 0.25 | Yes | 17 | No | No | No | Yes | No |
| 11 | Female | 57 | 20.5 | 30 | 0.375 | Yes | 41 | Yes | No | Yes | Yes | Yes |
| 12 | Female | 72 | 25.3 | 20 | 0.25 | Yes | 27 | No | No | No | No | No |
| 13 | Female | 64 | 24.5 | 25 | 0.5 | Yes | 57 | No | No | Yes | No | No |
| 14 | Female | 51 | 19.8 | 26 | 0.25 | Yes | 32 | Yes | No | Yes | No | Yes |
| 15 | Male | 65 | 20.7 | 20 | 0.675 | Yes | 14 | No | No | Yes | No | Yes |
| 16 | Female | 83 | 31.3 | 17 | 0.375 | Yes | 43 | No | No | No | Yes | No |
| 17 | Female | 61 | 26.6 |  | 0.5 | Yes | 40 | No | No | Yes | No | Yes |
| 18 | Male | 63 | 16.8 | 23 | 0.375 | Yes | 35 | Yes | No | Yes | Yes | No |
| 19 | Female | 63 | 17.6 |  | 0.25 | Yes | 37 | No | No | Yes | No | No |
| 20 | Female | 76 | 23.5 | 20 | 0.375 | Yes | 31 | No | No | Yes | Yes | No |
| 21 | Female | 75 | 21.6 | 29 | 0.25 | Yes | 25 | No | No | Yes | No | Yes |
| 22 | Female | 59 | 20.8 | 16 | 0.125 | No | 33 | No | No | Yes | No | No |
| 23 | Female | 43 | 24.2 |  | 0.375 | No | 98 | No | Yes | Yes | Yes | No |
| 24 | Male | 65 | 26.5 | 28 | 0.25 | No | 33 | No | No | Yes | No | No |
| 25 | Female | 62 | 21.5 | 19 | 0.25 | No | 94 | No | No | Yes | No | No |
| 26 | Female | 73 | 20.2 | 30 | 0.1875 | No | 89 | Yes | No | Yes | Yes | No |
| 27 | Female | 64 | 24.5 | 29 | 0.25 | No | 93 | No | No | Yes | No | No |
| 28 | Male | 58 | 25.4 | 33 | 0.375 | No | 99 | No | Yes | Yes | No | No |
| 29 | Male | 71 | 18.8 | 24 | 0.25 | No | 102 | Yes | Yes | No | Yes | No |
| 30 | Male | 50 | 25.8 | 35 | 0.375 | No | 104 | Yes | No | Yes | No | Yes |
| 31 | Female | 72 | 17.5 | 30 | 0.5 | No | 102 | No | No | No | No | Yes |
| 32 | Female | 72 | 21.4 | 8 | 0.375 | No | 84 | No | No | Yes | No | No |
| 33 | Male | 69 | 22.5 | 33 | 0.375 | No | 98 | Yes | No | Yes | No | No |
| 34 | Male | 60 | 24.2 | 26 | 0.25 | No | 97 | No | No | Yes | No | No |
| 35 | Male | 58 | 26.9 | 26 | 0.375 | No | 96 | No | No | Yes | No | No |
| 36 | Female | 58 | 22 | 15 | 0.25 | No | 94 | No | No | Yes | Yes | No |
| 37 | Female | 63 | 26.7 | 23 | 0.375 | No | 95 | No | No | Yes | No | No |
| 38 | Female | 56 | 21.2 | 20 | 0.5 | No | 95 | No | No | Yes | No | No |
| 39 | Male | 64 | 21.9 | 38 | 0.375 | No | 92 | No | No | No | No | No |
| 40 | Female | 62 | 25.9 | 25 | 0.375 | No | 92 | No | No | No | No | No |
| 41 | Female | 58 | 22.2 | 29 | 0.125 | No | 92 | 0 | No | Yes | No | Yes |
| 42 | Male | 43 | 19.3 | 23 | 0.25 | No | 93 | No | No | Yes | No | No |
| 43 | Female | 63 | 19.7 | 35 | 0.25 | No | 90 | No | No | No | No | No |
| 44 | Female | 65 | 19.1 | 18 | 0.375 | No | 84 | No | No | Yes | Yes | No |
| 45 | Male | 48 | 19.8 | 28 | 0.375 | No | 71 | No | No | Yes | No | Yes |
| 46 | Male | 51 | 21.1 | 26 | 0.25 | No | 91 | Yes | No | Yes | No | No |
| 47 | Female | 25 | 17.8 | 19 | 0.125 | No | 92 | No | No | No | Yes | No |
| 48 | Male | 36 | 22.8 | 20 | 0.25 | No | 92 | Yes | No | No | No | No |
| 49 | Female | 44 | 22.1 |  | 0.25 | No | 90 | Yes | No | No | Yes | No |
| 50 | Male | 44 | 22.3 | 24 | 0.375 | No | 89 | No | No | Yes | No | No |
| 51 | Female | 65 | 25.4 | 11 | 0.25 | No | 84 | No | No | Yes | No | No |
| 52 | Female | 80 | 19.3 |  | 0.5 | No | 79 | No | No | Yes | No | No |
| 53 | Female | 61 | 20.8 | 10 | 0.25 | No | 85 | No | No | Yes | No | No |
| 54 | Female | 68 | 18.7 | 28 | 0.125 | No | 84 | No | No | Yes | No | No |
| 55 | Female | 70 | 28.3 | 35 | 0.375 | No | 83 | No | No | Yes | Yes | No |
| 56 | Female | 70 | 22.1 | 28 | 0.25 | No | 83 | No | No | No | No | No |
| 57 | Male | 74 | 19.4 | 24 | 0.375 | No | 83 | No | No | Yes | No | No |
| 58 | Female | 77 | 25.7 | 27 | 0.375 | No | 82 | No | No | Yes | Yes | Yes |
| 59 | Female | 59 | 23.6 | 18 | 0.25 | No | 81 | No | No | No | No | No |
| 60 | Female | 71 | 23.1 | 12 | 0.25 | No | 80 | Yes | No | Yes | No | No |
| 61 | Female | 38 | 21.9 | 26 | 0.375 | No | 79 | No | No | No | No | No |
| 62 | Female | 30 | 21.1 | 27 | 0.125 | No | 79 | No | No | No | No | No |
| 63 | Female | 63 | 21.1 | 26 | 0.25 | No | 78 | No | No | No | No | No |
| 64 | Male | 32 | 21.1 | 30 | 0.375 | No | 77 | Yes | No | Yes | No | No |
| 65 | Male | 58 | 23 | 25 | 0.375 | No | 76 | No | No | Yes | No | No |
| 66 | Female | 75 | 20.8 | 22 | 0.375 | No | 67 | No | No | No | No | No |
| 67 | Male | 67 | 24.5 | 18 | 0.375 | No | 70 | Yes | No | No | No | Yes |
| 68 | Female | 64 | 19.5 | 14 | 0.25 | No | 76 | Yes | No | Yes | No | No |
| 69 | Female | 85 | 18.2 | 29 | 0.375 | No | 71 | Yes | No | No | No | Yes |
| 70 | Female | 64 | 19.7 | 24 | 0.25 | No | 71 | No | No | Yes | No | No |
| 71 | Male | 66 | 21.8 | 19 | 0.25 | No | 75 | No | No | Yes | Yes | No |
| 72 | Female | 72 | 26.8 | 29 | 0.25 | No | 66 | Yes | No | Yes | No | No |
| 73 | Male | 68 | 21.5 |  | 0.375 | No | 73 | No | No | Yes | No | No |
| 74 | Male | 74 | 17.8 | 18 | 0.25 | No | 72 | No | No | No | Yes | No |
| 75 | Female | 46 | 27.7 |  | 0.25 | No | 67 | Yes | No | Yes | Yes | No |
| 76 | Female | 72 | 20.9 | 22 | 0.125 | No | 69 | No | No | No | No | No |
| 77 | Female | 37 | 16.9 | 28 | 0.0625 | No | 70 | No | No | No | Yes | No |
| 78 | Male | 34 | 20 |  | 0.25 | No | 68 | No | No | Yes | No | No |
| 79 | Male | 48 | 21.5 | 25 | 0.5 | No | 69 | Yes | No | No | No | No |
| 80 | Female | 71 | 22.7 | 20 | 0.125 | No | 59 | No | No | No | No | No |
| 81 | Male | 24 | 23.4 | 29 | 0.125 | No | 67 | No | No | No | No | No |
| 82 | Female | 63 | 22.4 | 29 | 0.25 | No | 61 | No | Yes | No | No | No |
| 83 | Female | 65 | 18.6 | 14 | 0.125 | No | 65 | No | No | No | No | No |
| 84 | Female | 51 | 21 | 20 | 0.375 | No | 65 | No | No | No | Yes | No |
| 85 | Male | 48 | 20.3 | 20 | 0.375 | No | 57 | Yes | No | Yes | Yes | Yes |
| 86 | Male | 73 | 21.5 | 37 | 0.725 | No | 63 | No | No | No | No | No |
| 87 | Female | 67 | 20.1 | 19 | 0.25 | No | 63 | Yes | No | No | Yes | No |
| 88 | Male | 68 | 20.8 | 13 | 0.25 | No | 62 | No | No | No | No | No |
| 89 | Male | 71 | 23.4 | 15 | 0.25 | No | 62 | Yes | No | Yes | No | No |
| 90 | Male | 61 | 18.2 | 29 | 0.125 | No | 62 | Yes | No | Yes | No | No |
| 91 | Female | 65 | 17.5 | 17 | 0.25 | No | 62 | Yes | No | Yes | No | Yes |
| 92 | Male | 53 | 22.4 | 29 | 0.25 | No | 61 | Yes | No | No | No | No |
| 93 | Female | 71 | 20.2 | 20 | 0.125 | No | 61 | Yes | No | No | No | No |
| 94 | Female | 67 | 22 | 20 | 0.125 | No | 53 | No | No | Yes | Yes | Yes |
| 95 | Female | 64 | 20.5 | 25 | 0.25 | No | 58 | No | No | Yes | No | No |
| 96 | Male | 71 | 24.3 | 30 | 0.375 | No | 58 | No | No | Yes | No | No |
| 97 | Female | 59 | 22.6 | 19 | 0.125 | No | 58 | Yes | No | No | No | No |
| 98 | Male | 22 | 15.7 | 35 | 0.1875 | No | 57 | No | No | No | No | No |
| 99 | Female | 71 | 22.6 | 28 | 0.25 | No | 55 | No | No | Yes | No | No |
| 100 | Male | 71 | 23.7 |  | 0.375 | No | 40 | No | No | No | No | No |
| 101 | Female | 51 | 20.5 | 19 | 0.125 | No | 48 | Yes | No | Yes | No | No |
| 102 | Female | 51 | 20.3 | 17 | 0.25 | No | 52 | No | No | Yes | Yes | Yes |
| 103 | Male | 75 | 16.8 | 30 | 0.125 | No | 53 | No | No | No | No | No |
| 104 | Female | 68 | 18.8 |  | 0.375 | No | 51 | No | No | Yes | No | No |
| 105 | Female | 65 | 17.2 | 27 | 0.125 | No | 53 | Yes | No | Yes | No | Yes |
| 106 | Female | 68 | 20 | 21 | 0.125 | No | 50 | No | No | No | No | No |
| 107 | Female | 78 | 20.2 | 21 | 0.25 | No | 50 | No | No | Yes | No | No |
| 108 | Female | 48 | 21 | 19 | 0.125 | No | 46 | Yes | No | No | Yes | No |
| 109 | Male | 70 | 19.3 | 18 | 0.25 | No | 46 | Yes | No | Yes | Yes | No |
| 110 | Male | 48 | 32.8 | 35 | 0.25 | No | 41 | No | No | No | No | No |
| 111 | Male | 68 | 24.7 | 23 | 0.375 | No | 45 | No | No | Yes | No | No |
| 112 | Female | 82 | 19.1 | 34 | 0.125 | No | 45 | No | No | Yes | Yes | No |
| 113 | Female | 32 | 20.1 | 23 | 0.375 | No | 43 | No | No | Yes | Yes | No |
| 114 | Female | 65 | 22.4 | 21 | 0.25 | No | 43 | No | No | Yes | No | No |
| 115 | Female | 64 | 22.9 | 13 | 0.125 | No | 43 | No | No | No | No | No |
| 116 | Female | 71 | 16.4 |  | 0.125 | No | 42 | No | No | No | No | No |
| 117 | Female | 72 | 21.7 | 26 | 0.25 | No | 31 | No | No | Yes | No | No |
| 118 | Male | 71 | 26.5 |  | 0.375 | No | 30 | No | No | Yes | No | No |
| 119 | Male | 59 | 20.2 |  | 0.375 | No | 42 | No | No | Yes | No | No |
| 120 | Female | 55 | 25.6 | 21 | 0.25 | No | 40 | No | No | No | No | No |
| 121 | Female | 26 | 22.8 | 21 | 0.125 | No | 13 | No | No | Yes | No | No |
| 122 | Female | 85 | 17.4 | 16 | 0.1875 | No | 34 | No | No | No | Yes | No |
| 123 | Female | 63 | 26 | 30 | 0.25 | No | 40 | No | No | Yes | No | No |
| 124 | Male | 37 | 19.6 | 35 | 0.375 | No | 41 | No | No | Yes | No | Yes |
| 125 | Male | 37 | 25 | 19 | 0.125 | No | 38 | No | No | Yes | No | No |
| 126 | Female | 74 | 22.3 | 24 | 0.125 | No | 36 | No | No | Yes | No | Yes |
| 127 | Male | 74 | 20.5 | 35 | 0.375 | No | 36 | No | Yes | No | No | No |
| 128 | Female | 75 | 21.5 | 31 | 0.25 | No | 31 | No | No | Yes | No | Yes |
| 129 | Female | 70 | 22.6 | 26 | 0.25 | No | 34 | No | No | No | Yes | No |
| 130 | Female | 69 | 24.1 | 15 | 0.125 | No | 23 | No | No | No | No | No |
| 131 | Female | 68 | 25.9 | 30 | 0.25 | No | 33 | Yes | No | No | No | No |
| 132 | Female | 72 | 20.8 | 21 | 0.375 | No | 33 | No | No | No | No | No |
| 133 | Female | 64 | 21.7 | 21 | 0.25 | No | 33 | No | No | Yes | No | No |
| 134 | Male | 23 | 18.7 | 26 | 0.25 | No | 18 | Yes | No | No | No | No |
| 135 | Female | 75 | 20.3 | 25 | 0.25 | No | 25 | No | No | Yes | Yes | No |
| 136 | Male | 81 | 22.6 | 22 | 0.375 | No | 54 | Yes | Yes | No | No | Yes |
| 137 | Male | 77 | 21.7 | 14 | 0.125 | No | 30 | No | No | No | Yes | No |
| 138 | Male | 36 | 23.7 | 20 | 0.1875 | No | 12 | Yes | No | No | No | No |
| 139 | Male | 66 | 28.1 | 21 | 0.25 | No | 28 | Yes | No | No | No | No |
| 140 | Female | 58 | 28.4 |  | 0.25 | No | 27 | No | No | No | No | No |
| 141 | Male | 63 | 23.7 | 26 | 0.25 | No | 21 | No | No | Yes | No | No |
| 142 | Male | 50 | 23.9 | 11 | 0.125 | No | 24 | No | No | No | No | No |
| 143 | Male | 73 | 17.9 | 23 | 0.125 | No | 26 | No | No | No | No | No |
| 144 | Female | 76 | 22.9 | 31 | 0.1875 | No | 27 | No | No | Yes | No | No |
| 145 | Female | 78 | 17.1 | 22 | 0.375 | No | 93 | No | Yes | No | No | No |
| 146 | Male | 45 | 20.4 |  | 0.5 | No | 77 | No | No | No | No | Yes |
| 147 | Female | 57 | 21.7 |  | 0.25 | No | 78 | No | No | No | No | No |
| 148 | Male | 66 | 25.9 | 26 | 0.375 | No | 62 | No | No | No | No | No |
| 149 | Male | 74 | 24.1 |  | 0.5 | No | 56 | No | No | Yes | No | No |
| 150 | Female | 73 | 19.1 | 27 | 0.25 | No | 66 | No | No | No | No | No |
| 151 | Female | 71 | 20.8 | 31 | 0.25 | No | 66 | Yes | No | No | Yes | No |
| 152 | Female | 69 | 24.9 | 29 | 0.375 | No | 63 | No | No | Yes | Yes | No |
| 153 | Female | 66 | 30.4 |  | 0.25 | No | 61 | No | No | No | No | Yes |
| 154 | Male | 71 | 30.6 | 17 | 0.375 | No | 61 | No | No | Yes | No | No |
| 155 | Female | 57 | 23.7 |  | 0.25 | No | 59 | Yes | No | No | No | No |
| 156 | Female | 76 | 18.3 | 33 | 0.25 | No | 57 | No | No | Yes | No | Yes |
| 157 | Female | 70 | 25 | 25 | 0.1875 | No | 56 | Yes | No | Yes | No | No |
| 158 | Female | 60 | 24.5 | 19 | 0.25 | No | 42 | Yes | No | No | No | No |
| 159 | Male | 73 | 17.8 |  | 0.25 | No | 54 | No | No | Yes | No | Yes |
| 160 | Male | 64 | 24.9 | 13 | 0.125 | No | 20 | No | No | No | No | No |
| 161 | Male | 57 | 23.9 | 17 | 0.375 | No | 54 | No | No | Yes | No | No |
| 162 | Male | 59 | 20.2 | 35 | 0.125 | No | 52 | No | No | Yes | No | No |
| 163 | Male | 59 | 22.7 | 17 | 0.375 | No | 50 | No | No | Yes | No | No |
| 164 | Female | 80 | 18.9 | 26 | 0.25 | No | 50 | No | No | Yes | Yes | Yes |
| 165 | Female | 58 | 18.2 | 23 | 0.125 | No | 49 | No | No | Yes | No | No |
| 166 | Female | 82 | 21.7 | 32 | 0.125 | No | 25 | No | No | Yes | Yes | No |
| 167 | Female | 67 | 24 | 21 | 0.1875 | No | 48 | No | No | Yes | No | No |
| 168 | Male | 41 | 26.3 | 21 | 0.375 | No | 47 | No | No | Yes | No | No |
| 169 | Female | 66 | 21.6 | 20 | 0.25 | No | 48 | Yes | No | Yes | No | No |
| 170 | Male | 83 | 23.5 |  | 0.125 | No | 48 | No | No | No | No | No |
| 171 | Female | 32 | 27.1 | 19 | 0.25 | No | 48 | No | No | Yes | Yes | No |
| 172 | Female | 48 | 24.4 | 29 | 0.125 | No | 46 | No | No | Yes | Yes | No |
| 173 | Male | 68 | 30.1 | 19 | 0.375 | No | 45 | No | No | No | No | Yes |
| 174 | Female | 29 | 18.8 | 23 | 0.25 | No | 38 | Yes | No | No | Yes | No |
| 175 | Female | 43 | 20.6 | 27 | 0.25 | No | 42 | No | No | No | Yes | No |
| 176 | Male | 69 | 21.3 | 20 | 0.25 | No | 42 | No | No | No | Yes | No |
| 177 | Female | 70 | 21.7 | 21 | 0.25 | No | 27 | No | Yes | Yes | No | No |
| 178 | Female | 77 | 22.4 | 30 | 0.25 | No | 37 | Yes | No | Yes | Yes | No |
| 179 | Female | 75 | 22.4 |  | 0.25 | No | 29 | Yes | No | No | Yes | No |
| 180 | Female | 69 | 24.6 |  | 0.125 | No | 35 | No | No | Yes | No | Yes |
| 181 | Female | 78 | 24.6 | 31 | 0.25 | No | 35 | No | No | Yes | No | No |
| 182 | Female | 28 | 23.4 |  | 0.25 | No | 21 | No | No | Yes | No | No |
| 183 | Female | 54 | 19.2 |  | 0.25 | No | 31 | Yes | Yes | No | No | No |
| 184 | Male | 64 | 25.7 | 25 | 0.125 | No | 34 | No | No | Yes | No | No |
| 185 | Male | 56 | 27 | 26 | 0.25 | No | 32 | No | Yes | Yes | No | No |
| 186 | Male | 77 | 25.7 | 18 | 0.125 | No | 21 | Yes | No | No | No | No |
| 187 | Female | 76 | 17.1 | 21 | 0.125 | No | 26 | Yes | No | No | Yes | No |
| 188 | Male | 35 | 26.6 | 28 | 0.25 | No | 14 | No | Yes | Yes | Yes | No |
| 189 | Male | 58 | 21.7 |  | 0.125 | No | 28 | No | No | No | No | No |
| 190 | Male | 87 | 16.8 | 29 | 0.125 | No | 30 | No | No | Yes | No | No |
| 191 | Male | 53 | 21.2 | 25 | 0.25 | No | 11 | No | No | Yes | No | No |
| 192 | Female | 82 | 25 | 30 | 0.25 | No | 28 | No | No | No | No | No |
| 193 | Female | 63 | 16.4 |  | 0.25 | No | 30 | No | Yes | Yes | No | No |
| 194 | Female | 74 | 26.1 | 26 | 0.25 | No | 28 | No | No | Yes | No | Yes |
| 195 | Male | 41 | 19.1 | 23 | 0.125 | No | 23 | No | No | No | Yes | No |
| 196 | Male | 77 | 21.7 |  | 0.375 | No | 24 | Yes | No | No | No | Yes |
| 197 | Female | 81 | 21 |  | 0.125 | No | 24 | Yes | Yes | No | Yes | No |
| 198 | Female | 37 | 23.8 | 37 | 0.125 | No | 23 | Yes | No | Yes | Yes | No |
| 199 | Female | 36 | 20.2 | 29 | 0.25 | No | 22 | No | No | No | Yes | No |
| 200 | Female | 73 | 19.3 | 17 | 0.25 | No | 22 | Yes | No | No | No | No |
| 201 | Female | 44 | 21.2 | 20 | 0.125 | No | 10 | No | No | No | No | Yes |
| 202 | Male | 50 | 23.2 | 19 | 0.25 | No | 21 | No | No | Yes | No | No |
| 203 | Female | 48 | 19.6 | 21 | 0.125 | No | 23 | No | No | No | Yes | No |
| 204 | Female | 64 | 23.7 | 21 | 0.25 | No | 16 | Yes | No | Yes | No | No |
| 205 | Male | 61 | 20.1 | 10 | 0.25 | No | 21 | No | No | Yes | No | No |
| 206 | Female | 50 | 19.7 | 25 | 0.125 | No | 21 | Yes | No | No | Yes | No |
| 207 | Female | 39 | 19.4 | 28 | 0.25 | No | 21 | No | No | No | Yes | No |
| 208 | Female | 47 | 18.4 | 33 | 0.25 | No | 20 | Yes | No | No | Yes | No |
| 209 | Male | 76 | 14.5 | 15 | 0.25 | No | 11 | No | No | No | No | No |
| 210 | Male | 36 | 25.7 | 11 | 0.25 | No | 2 | No | No | No | No | No |
| 211 | Female | 65 | 21.8 | 21 | 0.25 | No | 20 | No | No | No | No | No |
| 212 | Female | 64 | 19.9 |  | 0.125 | No | 20 | No | No | No | No | No |
| 213 | Male | 67 | 22.6 |  | 0.125 | No | 18 | No | No | No | No | No |
| 214 | Female | 72 | 20.9 | 33 | 0.0625 | No | 12 | No | No | No | No | No |
| 215 | Male | 45 | 25.5 |  | 0.25 | No | 17 | Yes | No | Yes | No | Yes |
| 216 | Male | 24 | 37.6 | 28 | 0.25 | No | 16 | Yes | No | Yes | No | No |
| 217 | Male | 54 | 24.7 |  | 0.25 | No | 12 | No | No | No | No | No |
| 218 | Female | 52 | 21.3 | 31 | 0.25 | No | 5 | No | No | No | No | No |
| 219 | Female | 68 | 30 | 27 | 0.125 | No | 4 | No | No | No | No | No |
| 220 | Female | 75 | 24.7 | 18 | 0.125 | No | 15 | No | No | No | No | No |
| 221 | Male | 47 | 23.3 | 21 | 0.25 | No | 10 | No | No | No | No | No |
| 222 | Female | 46 | 29.7 | 23 | 0.125 | No | 5 | No | No | No | Yes | No |
| 223 | Female | 71 | 23.3 | 26 | 0.1875 | No | 16 | No | No | Yes | Yes | No |
| 224 | Female | 26 | 20.8 | 32 | 0.125 | No | 23 | No | No | No | No | No |
| 225 | Male | 12 | 16.6 | 34 | 0.03125 | No | 6 | No | No | No | Yes | No |
| 226 | Male | 69 | 25.4 | 19 | 0.125 | No | 5 | No | No | No | No | No |
| 227 | Female | 61 | 23.5 | 13 | 0.125 | No | 2 | No | No | No | No | No |
| 228 | Male | 56 | 27 | 29 | 0.25 | No | 11 | No | No | No | No | No |
| 229 | Female | 36 | 24.1 | 23 | 0.25 | No | 25 | No | Yes | No | No | No |
| 230 | Male | 83 | 25.8 |  | 0.25 | No | 8 | No | No | No | No | Yes |
| 231 | Male | 33 | 19.7 | 23 | 0.125 | No | 2 | No | No | No | No | No |
